# Pediatric Drug Safety Surveillance in FDA-AERS: A Description of Adverse Events from GRiP Project

**DOI:** 10.1371/journal.pone.0130399

**Published:** 2015-06-19

**Authors:** Sandra de Bie, Carmen Ferrajolo, Sabine M. J. M. Straus, Katia M. C. Verhamme, Jan Bonhoeffer, Ian C. K. Wong, Miriam C. J. M. Sturkenboom, GRiP network

**Affiliations:** 1 Department of Medical Informatics, Erasmus University Medical Center, Rotterdam, the Netherlands; 2 Medicines Evaluation Board, Utrecht, the Netherlands; 3 Experimental Medicine Department, Pharmacology Section, Campania Regional Center of Pharmacovigilance and Pharmacoepidemiology, Second University of Naples, Naples, Italy; 4 Brighton Collaboration Foundation, Basel, Switzerland; 5 University Children’s Hospital Basel, University of Basel, Basel, Switzerland; 6 Centre for Safe Medication Practice and Research, Department of Pharmacology and Pharmacy, University of Hong Kong, Hong Kong, China; 7 Department of Epidemiology, Erasmus University Medical Center, Rotterdam, the Netherlands; Mario Negri Institute for Pharmacology Research, ITALY

## Abstract

Individual case safety reports (ICSRs) are a cornerstone in drug safety surveillance. The knowledge on using these data specifically for children is limited. We studied characteristics of pediatric ICSRs reported to the US Food and Drug Administration (FDA) Adverse Event Reporting System (FAERS). Public available ICSRs reported in children (0–18 years) to FAERS were downloaded from the FDA-website for the period Jan 2004-Dec 2011. Characteristics of these ICSRs, including the reported drugs and events, were described and stratified by age-groups. We included 106,122 pediatric ICSRs (55% boys and 58% from United States) with a median of 1 drug [range 1–3] and 1 event [1–2] per ICSR. Mean age was 9.1 years. 90% was submitted through expedited (15-days) (65%) or periodic reporting (25%) and 10% by non-manufacturers. The proportion and type of pediatric ICSRs reported were relatively stable over time. Most commonly reported drug classes by decreasing frequency were ‘*nervous system drugs*’ (58%), ‘*antineoplastics*’ (32%) and ‘*anti-infectives*’ (25%). Most commonly reported system organ classes were ‘*general*’ (13%), ‘*nervous system*’ (12%) and ‘*psychiatric*’ (11%) disorders. Duration of use could be calculated for 19.7% of the reported drugs, of which 14.5% concerned drugs being used long-term (>6 months). Knowledge on the distribution of the drug classes and events within FAERS is a key first step in developing pediatric specific methods for drug safety surveillance. Because of several differences in terms of drugs and events among age-categories, drug safety signal detection analysis in children needs to be stratified by each age group.

## Introduction

The limited knowledge about the effects of drugs in children has boosted initiatives by the World Health Organisation (WHO) and triggered new legislation in recent years [1, 2]. The Global Research in Paediatrics Network of Excellence (GRiP) is an EU-funded consortium, which aims to implement an infrastructure facilitating the development and safe use of medicines in children. This entails the development of a comprehensive educational programme and integrated use of existing research capacity, whilst reducing the fragmentation and duplication of efforts [3, 4].

Post-marketing drug safety surveillance using spontaneous reporting systems is essential in studying drug safety [5]. An important part of the GRiP project is evaluating current and developing new methodology for post-marketing drug safety studies specifically for the pediatric population. Typical large spontaneous reporting systems include VigiBase of the WHO Uppsala Monitoring Center (WHO-UMC), the Adverse Event Reporting System (AERS), maintained by United States (US) Food and Drug Administration (FDA-AERS, FAERS), the Vaccine Adverse Effect Reporting System (VAERS), maintained by FDA and CDC (Centers for Disease Control and Prevention), and EudraVigilance of the European Medicines Agency (EMA) [6–9].

While these spontaneous reporting databases were predominantly used by regulatory authorities to monitor drug safety and to perform safety signal detection, these data are increasingly available for research purposes. The FDA databases offer publicly downloadable datasets [10, 11], while the EMA published their access policy for EudraVigilance in 2011 [12], and WHO-UMC is preparing summary VigiBase data to be made accessible via their website [13]. Understanding the structure and scope of these datasets and their respective strengths and limitations is essential for their correct use and interpretation and a first and important step for evaluating current and developing new methodology. In 2011 an overview of pediatric ICSRs reported to WHO-UMC was published [14]. Published descriptions on the pediatric reports within AERS include the number of reports, their outcome, and the most frequently reported drugs [15–17]. However, studies rarely reported on the reported adverse events within FAERS.

In the current study we aimed to describe the pediatric ICSRs as reported within FAERS. Specific attention was given to describing adverse events occurring within age-categories and after long-term drug use or with delayed onset after cessation of treatment.

## Methods

### Data source

FAERS is a database that contains information on adverse event and medication error reports submitted to FDA. It is a passive surveillance system that relies on voluntary reporting by healthcare professionals and consumers, as well as required reporting by pharmaceutical manufacturers. FAERS includes spontaneous reports from US sources; serious and unlabelled spontaneous reports from non-US sources; and serious, unlabelled, and attributable post-marketing clinical trial reports from all sources [10].

FAERS data is publicly available and files containing the raw data of individual case safety reports (ICSRs) as contained within the database are available [10]. The information include: patient demographic and administrative information; drug/biologic information; preferred terms of MedDRA (Medical Dictionary for Regulatory Activities) of the events; patient outcomes for the event; report sources for the event; therapy start dates and end dates; and indications of use (diagnosis) for the reported drugs.

### Data preparation

We extracted all ICSRs for the period January 2004 till December 2011 and included all ICSRs on children, aged 0 to <18 years. We excluded the following reports from the analyses: adults reports (≥18 years); reports with missing age or event; duplicate reports (e.g. in a follow-up report, were only included once). All reported events are coded in preferred terms of MedDRA. To facilitate high level descriptive we recoded the reported terms to a single System Organ Classes (SOCs) of MedDRA. The reported drugs are described either as a valid trade name or as unstructured narrative. As far as possible the reported drug names were recoded to Anatomical Therapeutic Chemical (ATC) drug classes using drug dictionaries [18–21]. This recoding was possible for >90% of the reported drugs reported in the selected ICSRs. The entries for which recoding was not possible included reports without a specified drug name, spelling errors. For the analyses on type of reported drugs and unique combination drug/event, only those records with a known ATC-code were included.

### Data analysis

Each of the included ICSRs was classified by the age at time of the event, sex, number of drugs and number of events. Results were stratified by age-categories in which age at onset was stratified based on the ICH (International Conference On Harmonisation) age-groups: neonates (≤27 days), infants (28 days-≤23 months), children (2-≤11 years), and adolescents (12-≤17 years) [22].

The role of the reported drugs, being either primary suspect, secondary suspect, concomitant or interacting was provided [23]. The most frequently reported drugs and events were described. In addition, the reported events were stratified with respect to the outcome of the event. The outcome was registered in terms of the seriousness criteria: death, life-threatening, hospitalization (initial or prolonged), disability, congenital anomaly, requiring intervention to prevent permanent impairment or damage or other. Using the primary suspect and secondary suspect drugs only, the most frequent reported drug-event combinations were described.

For those drugs for which the starting date of the drug and the date of the event were known, the time to event was calculated. An event occurred after long-term use if it occurred at least 6 months after starting of therapy [24]. We also studied delayed events. For those records with a known stopping date of therapy and date of the event, the type of reported events occurring >3 months after drug cessation were compared with the type of events occurring during drug use.

Characteristics of the ICSRs were compared using chi-square to compare proportions and either students-t test, if variable was normally distributed or Mann-Whitney tests if the variable was not normally distributed to compare means. A *p*-value <0.05 was considered to be statistical significant.

## Results

**Fig 1:**
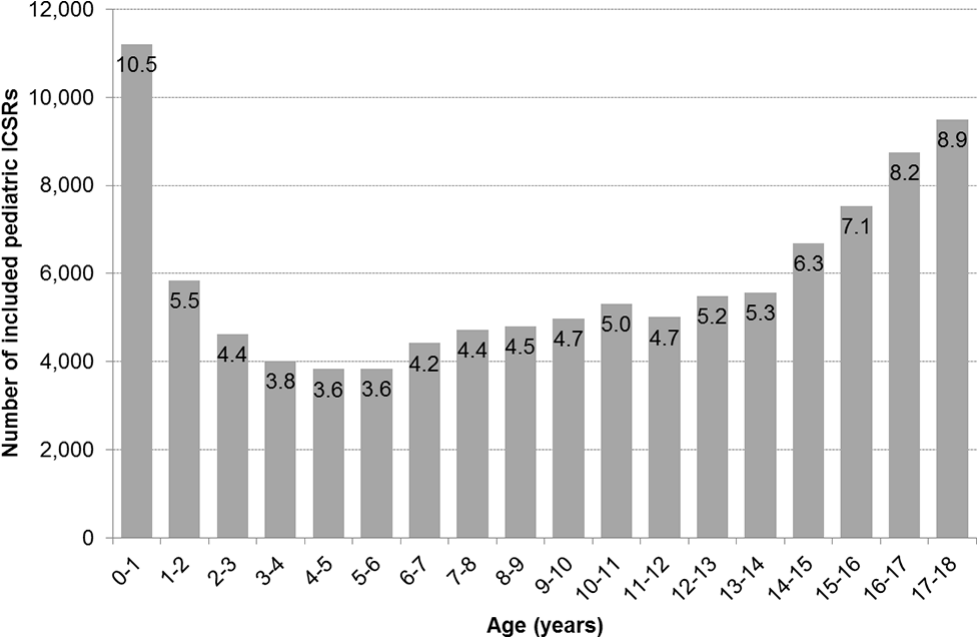
Number of reported ICSRs by age. Within the bars the proportion of ICSRs within this age-stratum of the total reported pediatric ICSRs is given.

The overall publicly available dataset of FAERS included 3,691,417 ICSRs. Among them, 3,585,295 ICSRs were excluded, 2,311,727 of which concerned adults, 1,234,941 with age and/or event missing, and 38,627 duplicates. Accordingly, 106,122 (2.9%) ICSRs occurred in children <18 years and were included in the analyses. The mean age of the children in these reports was 9.1 years. 10.5% of the ICSRs were on children up to one year of age, after which this decreased to 3.6% at 4 to 5 years of age and gradually increased again to 8.9% at 17–18 years of age (Fig 1). The majority of the ICSRs (54.5%) were reported for boys; reports for boys exceeded those for girls up to the age of 11 years (54.1–59.9%) and this reversed from the age of 12 years onwards (47.7%), the mean age in the female reports was higher than for male reports (*p*<0.001). The ICSRs comprised a total of 236,491 drug records (median 1 drug/ICSR). The ICSRs comprised a total 397,220 event records (median 1 event/ICSR) (Table 1). The outcome in terms of seriousness criteria was: 33% hospitalization (initial or prolonged); 12% death; 3% life-threatening; 3% disability; 2% congenital anomaly; 1% required intervention to prevent permanent impairment or damage; 31% other and was missing in 15% (data not shown).

**Table 1:** Distribution of pediatric ICSRs (N = 106,122) within FAERS according to age-category.

	Total	≤ 27 days	28 days-23 months	2–11 years	12–17years
	N = 106,122 (%)	N = 4,717 (4.4%)	N = 16,096 (15.2%)	N = 47,248 (44.5%)	N = 38,061 (35.9%)
Males	54,768 (54.5%)	2,114 (54.1%)	7,921 (55.3%)	27,075 (59.9%)	17,658 (47.7%)
Mean age (95%CI)	9.1 (9.0–9.1)				
Reported drugs	236,491	12,180 (5.2%)	34,575 (14.6%)	103,988 (44.0%)	85,748 (36.3%)
Drugs/ICSR [median (IQR)]	1 (1–3)	1 (1–3)	1 (1–3)	1 (1–3)	1 (1–3)
Reported events	397,220	21,265 (5.4%)	59,306 (14.9%)	173,395 (43.7%)	143,254 (36.1%)
Events/ICSR [median (IQR)]	1 (1–1)	1 (1–2)	1 (1–2)	1 (1–1)	1 (1–1)

**Fig 2:**
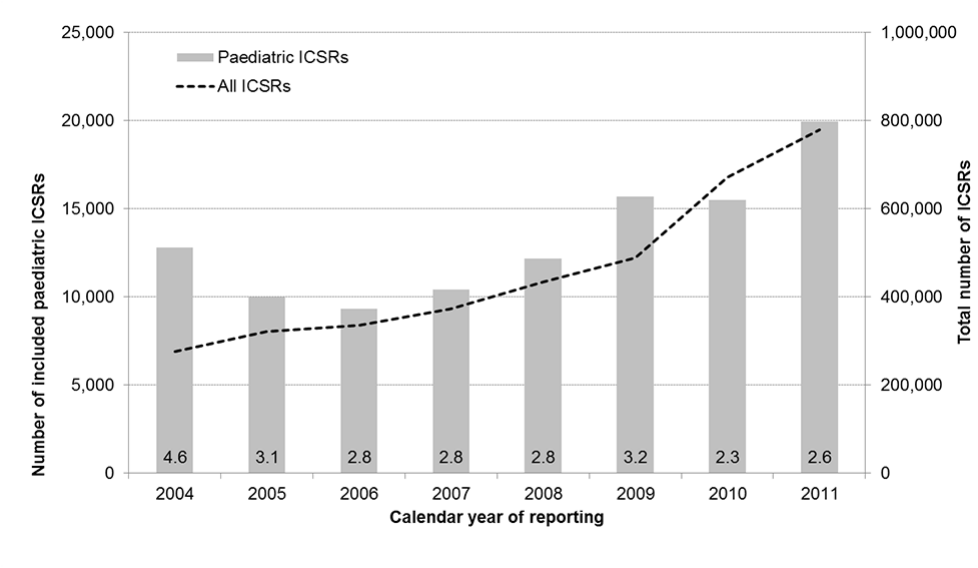
Number of reported ICSRs over time. The number of included pediatric ICSRs is plotted on the left y-axis. The total number of ICSRs within the database is plotted on the right y-axis. Within the bars the proportion of pediatric ICSRs of the total reported ICSRs is given.

The number of ICSRs reported by calendar year is increasing, with a small dip in 2010 (Fig 2).

Of the included ICSRs, 10.0% (N = 10,576) concerned *direct* reporting, defined as ICSRs being voluntary submitted by “non-manufacturers”. The majority of the ICSRs were submitted by manufacturers; 64.9% (N = 68,886) were expedited reports and 25.1% (N = 26,660) were periodic reports. The reporter was a physician in 32.0% of the ICSRs, a consumer in 24.9% and another health professional in 20.0%. The majority of the reports originated from the US (58%), followed by Japan (7.0%) and the United Kingdom (6.6%) (Table 2).

**Table 2:** Main characteristics of pediatric ICSRs (N = 106,122) within FAERS.

Type of report	N of ICSRs (%)
Direct reporting	10,576 (10.0%)
Expedited reports (“15 day reports”)	68,886 (64.9%)
Periodic reports	26,660 (25.1%)
**Reporter**	
Physician	33,990 (32.0)
Consumer/non-health professional	26,378 (24.9)
Other health professional	21,193 (20.0)
Pharmacist	6,159 (5.8)
Lawyer	1,301 (1.2)
Unspecified	17,101 (16.1)
**Initial source**	
Foreign	10,290 (9.7%)
Study	164 (0.2%)
Literature	882 (0.8%)
Consumer	10,123 (9.5%)
Health Professional	11,196 (10.6%)
User Facility	14 (0.0%)
Company representative	3,964 (3.7%)
Distributor	229 (0.2%)
Other	554 (0.5%)
Unknown	68,706 (64.7%)
**Country**	
United States	50,625 (47.7%)
Japan	6,119 (5.8%)
United Kingdom	5,722 (5.4%)
France	4,656 (4.4%)
Germany	2,758 (2.6%)
Unknown	18,827 (17.7%)

**Fig 3:**
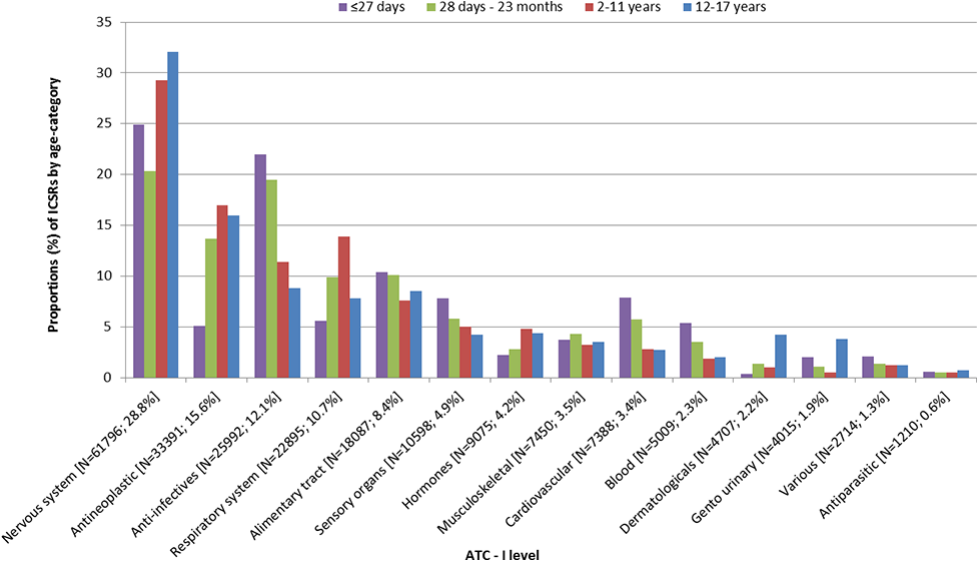
Proportions of reported drugs by anatomical main group. Distribution of the number of reported drugs over anatomical main group (1st level ATC),stratified by age-categories. The reported ATC classes are presented at the X-axis including the number of reports and the percentage of total. On the y-axis the distribution of the age-categories within each ATC class is presented, counting up to 100%. Within the bars the proportion of this ATC class within the total number of reported drugs within the specified age-category is presented. Only those drugs with a recoded ATC code are included (N = 214,327).

Among the included ICSRs, 35% of reported drugs were indicated as primary suspected, 21% as secondary suspected and the remaining drugs as concomitant or interacting (44%). ‘*Nervous system drugs*’, ‘*antineoplastics*’ and ‘*anti-infective agents*’ were the most frequently reported drug class in all age-categories (Fig 3). Specifically, among ‘*nervous system drugs*’, mainly ‘*antiepileptic drugs*’ and ‘*analgesics*’ were reported in the youngest children and drugs to treat ADHD in the older children (Table 3). ‘*Anti-infectives*’ were an important group of the reported drugs for the youngest children, covering 22% of the reported drugs in children <27 days of age (‘*antiretroviral drugs’* and ‘*antibiotics’*) and 20% of the drugs in children aged 28 days to 23 months (‘*specific* i*mmunoglobulins’* and ‘*antibiotics’*). In the older children the *‘anti-infectives’* covered a smaller proportion of the drugs and *‘antineoplastic drugs’* became of more importance; 17% in children aged 2 to 11 years and 16% in children aged 12 to 17 years.

**Table 3:** Most frequently reported drugs.

≤27 days	N (%)	28 days—23 months	N (%)	2–11 years	N (%)	12–17 years	N (%)	Total	N (%)
Zidovudine	310 (2.9%)	Palivizumab	1025 (3.3%)	Atomoxetine	4680 (4.9%)	Isotretinoin	2634 (3.4%)	Atomoxetine	6597 (3.1%)
Vitamines	229 (2.1%)	Paracetamol combinations	905 (3.0%)	Methylphenidate	3557 (3.8%)	Atomoxetine	1897 (2.4%)	Methylphenidate	5224 (2.4%)
Ampicillin	170 (1.6%)	Paracetamol	746 (2.4%)	Montelukast	2067 (2.2%)	Methylphenidate	1637 (2.1%)	Paracetamol	3704 (1.7%)
Valproic acid	160 (1.5%)	Ibuprofen	611 (2.0%)	Fluticasone	1705 (1.8%)	Paracetamol	1304 (1.7%)	Methotrexate	3192 (1.5%)
Dopamine	140 (1.3%)	Ranitidine	501 (1.6%)	Methotrexate	1669 (1.8%)	Methotrexate	1207 (1.5%)	Montelukast	3015 (1.4%)
Furosemide	138 (1.3%)	Mitoxantrone	372 (1.2%)	Paracetamol	1546 (1.6%)	Lamotrigine	1165 (1.5%)	Valproic acid	2887 (1.3%)
Gentamicin	137 (1.3%)	Furosemide	342 (1.1%)	Valproic acid	1524 (1.6%)	Drospirenone and estrogen	1077 (1.4%)	Ibuprofen	2881 (1.3%)
Lamivudine	136 (1.3%)	Amoxicillin	328 (1.1%)	Somatropin	1392 (1.5%)	Infliximab	1038 (1.3%)	Isotretinoin	2810 (1.3%)
Insulin	130 (1.2%)	Amoxicillin / clavulanic acid	310 (1.0%)	Ibuprofen	1331 (1.4%)	Aripriprazole	1012 (1.3%)	Fluticasone	2590 (1.2%)
Nitric oxide	130 (1.2%)	Choline salicylate	309 (1.0%)	Salbutamol	1260 (1.3%)	Valproic acid	940 (1.2%)	Lamotrigine	2483 (1.2%)

**Fig 4:**
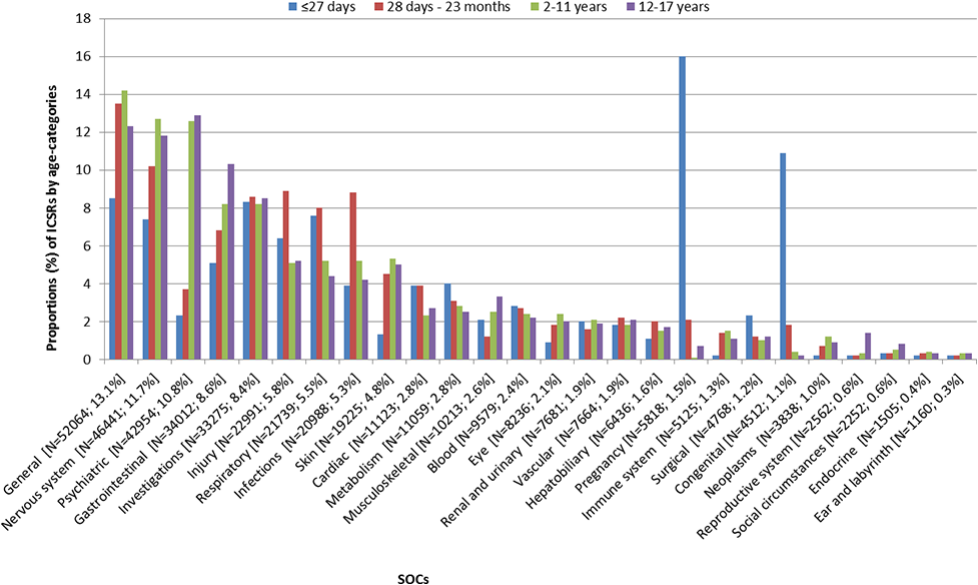
Proportions of reported events by system organ classes. Distribution of the number of reported events over system organ classes (SOCs), stratified by age-categories. The reported SOCs are presented at the X-axis including the number of reports and the percentage of total. On the y-axis the distribution of the age-categories within each SOC is presented, counting up to 100%. Within the bars the proportion of this SOC within the total number of reported events within the specified age-category is presented.

In the Fig 4 the reported events are stratified by their System Organ Classes (SOCs) and by age-categories. The ten most frequently reported events are presented in the Table 4. The reported events were most frequently situated in the SOCs *‘general disorders and administration site conditions’* (13%) (e.g. *‘vomiting’* and *‘pyrexia’*), *‘nervous system disorders’* (12%) (e.g. *‘convulsion’* and *‘headache’*), and *‘psychiatric disorders’* (11%) (e.g.*‘abnormal behaviour’* and *‘aggression’*). In the youngest group of children *‘pregnancy*, *puerperium and perinatal conditions’* (16%) and *‘congenital*, *familial and genetic disorders’* (11%) covered a large part of the reported events (e.g. ‘*drug exposure during pregnancy*’, ‘*premature baby*’ and ‘*maternal drugs affecting foetus*’). The proportion of *‘psychiatric disorders’* increased with age from 5% at ≤27 days of age to 13% at 12 to 17 years of age. Also reports of *‘nervous system disorders*’ increased with age from 7% at ≤27 days of age to 12–13% at 2–17 years of age.

**Table 4:** Most frequently reported events.

≤27 days	N (%)	28 days—23 months	N (%)	2–11 years	N (%)	12–17 years	N (%)	Total	N (%)
Drug Exposure During Pregnancy	1,350 (6.3%)	Pyrexia	1,068 (1.8%)	Vomiting	2,818 (1.6%)	Vomiting	1,878 (1.3%)	Vomiting	5,827 (1.5%)
Premature Baby	562 (2.6%)	Vomiting	1,046 (1.8%)	Pyrexia	2,425 (1.4%)	Headache	1,747 (1.2%)	Pyrexia	4,880 (1.2%)
Maternal Drugs Affecting Foetus	484 (2.3%)	Convulsion	895 (1.5%)	Drug Ineffective	2,394 (1.4%)	Nausea	1,641 (1.1%)	Convulsion	4,720 (1.2%)
Neonatal Disorders	381 (1.8%)	Accidental Drug Intake By Child	789 (1.3%)	Convulsion	2,334 (1.3%)	Depression	1,581 (1.1%)	Drug Ineffective	4,392 (1.1%)
Drug Withdrawal Neonatal Syndrome	333 (1.6%)	Diarrhoea	645 (1.1%)	Abnormal Behaviour	2,261 (1.3%)	Convulsion	1,399 (1.0%)	Headache	3,531 (0.9%)
Caesarean Section	328 (1.5%)	Accidental Exposure	622 (1.0%)	Aggression	1,755 (1.0%)	Drug Ineffective	1,365 (1.0%)	Abnormal Behaviour	3,264 (0.8%)
Maternal Exposure During Pregnancy	158 (0.7%)	Drug Ineffective	548 (0.9%)	Headache	1,733 (1.0%)	Pyrexia	1,322 (0.9%)	Nausea	3,093 (0.8%)
Neonatal Respiratory Distress Syndrome	140 (0.7%)	Somnolence	544 (0.9%)	Somnolence	1,392 (0.8%)	Suicidal Ideation	1,140 (0.8%)	Somnolence	2,903 (0.7%)
Drug Exposure Via Breast Milk	134 (0.6%)	Product Quality Issue	541 (0.9%)	Nausea	1,330 (0.8%)	Overdose	1,118 (0.8%)	Overdose	2,713 (0.7%)
Patent Ductus Arteriosus	122 (0.6%)	Overdose	540 (0.9%)	Product Quality Issue	1,238 (0.7%)	Suicide Attempt	1,113 (0.8%)	Aggression	2,710 (0.7%)

The number of reported drug-event pairs was calculated using the primary and secondary suspected drugs only. The number of unique drug-event combinations was 548,640 overall and within the age groups: 3,274 (≤27 days); 21,356 (28 days-23 months); 146,094 (2–11 years); 129,699 (12–17 years) (Table 5). Duration of drug use could be calculated for 63,311 drug records (26.8%). The median duration of use was 10 days (range 0–6,209). The starting and event date were equal in 28.4% of the records, 19.1% were reported after 1–7 days since starting. Time to event was 8–30 days in 14.4% of the records, 31–182 days in 17.8% and >182 days (defined as long-term use) in 20.2% of the records. The proportion of drugs being used long-term increased with age: 7.8% (28 days-23 months); 22.0% (2–11 years) and 24.1% (12–17 years). Within drug groups (i.e. ATC I level) the main differences between long-term use and short-term use concerned drugs belonging to *‘systemic hormonal preparations’* [10.1% of ADRs due to long-term use vs. 2.9% of ADRs due to short-term use; ratio: 3.5 (95% CI: 1.8–6.1)] and to *‘alimentary drugs’* [10.8% vs. 5.8%; 3.9 (2.1–6.8)]. On the contrary, the drug groups more reported after their short-term than long-term use were *‘anti-infectives*’ [18.0% for short-term use vs. 7.1% for long-term use; ratio: 2.5 (1.6–3.9)], *‘musculoskeletal system drugs’* [4.4% vs. 1.6%; 2.8 (0.8–6.0)], and *‘sensory organ agents’* [4.5% vs. 2.2%; 2.0 (0.6–4.4)]. The most frequently reported drugs after long-term use were *‘somatropin’* (N = 955; 7.5%), *‘atomoxetine’* (N = 507; 4.0%), and *‘methylphenidate’* (N = 462; 3.6%).

**Table 5:** Most frequently reported drug-ADR combinations (Primary and secondary suspected drugs only).

≤27 days	N = 3,274 (%)	28 days—23 months	N = 21,356 (%)	2–11 years	N = 146,094 (%)	12–17 years	N = 129,699 (%)	Total	N = 548,640 (%)
Buprenorphine–Drug withdrawal syndrome neonatal	32 (1.0%)	Valproate—Drug exposure during pregnancy	84 (0.4%)	Atomoxetine–Prescribed overdose	473 (0.3%)	Isotretinoin—Depression	472 (0.4%)	Isotretinoin—Depression	669 (0.1%)
Heparin—Maternal drugs affecting foetus	29 (0.9%)	Fluoxetine- Drug exposure during pregnancy	49 (0.2%)	Atomoxetine–Drug Ineffective	462 (0.3%)	Isotretinoin-Inflammatory bowel disease	337 (0.3%)	Atomoxetine–Drug Ineffective	664 (0.1%)
Heparin—Premature baby	21 (0.6%)	Valproate–Foetal anticonvulsant syndrome	40 (0.2%)	Atomoxetine–Abnormal behaviour	396 (0.3%)	Isotretinoin-Colitis Ulcerosa	257 (0.2%)	Atomoxetine–Prescribed overdose	654 (0.1%)
Levetiracetam—Maternal drugs affecting foetus	17 (0.5%)	Olanzapine—Drug exposure during pregnancy	39 (0.2%)	Methylphenidate–Product quality issue	393 (0.3%)	Isotretinoin-Crohn's disease	234 (0.2%)	Atomoxetine–Abnormal behaviour	579 (0.1%)
Heparin-Caesarean section	14 (0.4%)	Fentanyl–Accidental drug intake by child	38 (0.2%)	Atomoxetine–Somnolence	356 (0.2%)	Isotretinoin-Suicidal ideation	225 (0.2%)	Methylphenidate–Product quality issue	573 (0.1%)

Presented proportion are based on the total number of “primary suspect” and secondary suspect” reported drugs. The number of unique drug-event combinations was 180,100 and within the age groups: 2,606 (0–27 days); 14,800 (28 days-23 months); 62,788 (2–11 years); 59,101 (12–17 years).

In terms of SOCs, *‘neoplasms benign and malignant’* [2.1% vs. 0.5%; ratio: 4.2 (0.7–13.1)], *‘infections and infestations’* [7.0% vs. 4.8%; 1.5 (0.6–2.9)] and *‘musculoskeletal*, *connective tissue and bone disorders’* [3.8% vs. 2.3%; 1.7 (0.6–4.2)] were more often reported after long-term use, but the differences were no significant. The most frequently reported events after long-term use were *‘vomiting’* (N = 415; 1.2%), *‘convulsion’* (N = 412; 1.2%), and *‘pyrexia’* (N = 389; 1.2%). For 47,301 drug records (20.0%) the time between ceasing of therapy and occurrence of the event was known. The event occurred prior to stopping of the drug in 42.1% of the records, on the day of stopping of the drug in 31.5% and after stopping of therapy in 26.4%. Of the drugs occurring after stopping therapy, 45.2% occurred within 1–7 days, 27.0% occurred within 8–30 days, 11.3% within 31–90 days, 5.4% within 91–182 days, 5.1% within 183–365 days and 6.0% after 365 days. When comparing the drug classes reported >90 days of ceasing of therapy with those drug classes reported during drug use, the largest differences, with higher proportion for delayed effects, were present for *‘antineoplastic and immunomodulating agents’* [26.6% vs. 9.9%; ratio: 2.7 (1.8–3.9)], and *‘dermatologicals’* [10.8% vs. 5.1%; 2.1 (1.1–3.7)], while no significant differences were for the other drug groups. The most frequently reported drugs after delayed use (>3 months) were *‘isotretinoin’* (N = 184; 8.8%), *‘palivizumab’* (N = 95; 4.5%), and *‘infliximab’* (N = 75; 3.6%). *‘Neoplasms benign and malignant’* [2.9% vs. 0.6%; ratio: 4.8 (1.3–13.6)], *‘gastrointestinal disorders’* [14.8% vs. 9.8%; 1.5 (0.9–2.5)], and *‘infections and infestations’* [7.5% vs. 4.6%; 1.6 (0.8–3.3)] were the most frequently SOCs reported 3 months after stopping. The most frequently reported delayed events were *‘Crohn’s disease’* (N = 106; 1.7%), *‘inflammatory bowel disease’* (N = 98; 1.6%), and *‘depression’* (N = 89; 1.4%).

## Discussion

We explored the characteristics of the reports in children and adolescents in the US FAERS database, including number and type of ICSRs, their outcome and the most frequently implicated drugs and events. The largest proportion of ICSRs was reported for boys until the age of 11 after which it reversed to girls. These findings, in line with previous researches on other spontaneous reporting systems [14, 25], can be explained by several factors. On one hand, some childhood diseases, i.e. asthma [26], certain infections [27], ADHD [28], requiring a treatment with medications commonly implicated in ICSRs, occur more frequently among younger boys than younger girls; afterwards, the incidence changes during the adolescence, mainly for asthma and urinary tract infections, with higher occurrence among girls than boys. On the other hand, younger boys and older girls might be physiologically vulnerable to adverse events. Irrespective of the gender, we found an overall increase of the absolute number of reports with age increasing that could be attributed to a greater exposure to the medications in adolescents [29].

Pediatric adverse events reported to FAERS have been previously investigated by Johann-Liang *et al*., with focus on potential change of the trend of spontaneous reporting following the implementation of the Best Pharmaceuticals for Children Act (BPCA) in 2002 [15]. Then, they investigated the differences in children and adults with regard to drug safety monitoring and reporting. Conversely, we aimed to analyze the distribution of the adverse events reported in children in terms of age-categories. We observed an expected variation between age-categories in the type of reported drug and event, consistently with drug prescribing pattern in US. As example, the increase of the proportion of ICSRs involving nervous system drugs after 2 years of age is explained by the highest rates of prescriptions of ADHD therapies (i.e. *‘methylphenidate’*) in adolescents [30]. As a consequence, we might explain the observed increase of adverse events like as nervous system and psychiatric disorders in adolescents.

The pediatric ICSRs coming from US were previously described by Star *et al*., through the analysis of VigiBase [14]. Although US reports make up the largest proportion of the ICSRs both within FAERS (58%) and within VigiBase (39%), striking differences between the datasets are present. First, the type of reporters differed. While more than half of the ICSRs of VigiBase were reported by physicians, only a third of the FAERS ICSRs were reported by physicians. The most notable difference was for consumer reports; 24.9% of the FAERS reports versus 4.3% within VigiBase. This difference might be due to different time-periods; consumer reporting is increasing in latest years [31]. Second, only a small proportion of the FAERS ICSRs concerned reporting by non-manufacturers. The majority was either reported as part of expedited reporting (65%) or as part of periodic reporting. Earlier it was shown that the US reports within VigiBase are mainly reported by manufacturers, while these form only a small proportion of the reports from the other continents [7]. Third, the reported drug groups and events differed. VigiBase reports more often concerned *‘anti-infectives’* and *‘dermatological drugs’*, while within FAERS *‘neurological drugs’* and *‘antineoplastic drugs’* were most frequently reported. This also reflects utilization differences between the US and Europe, with high rates of prescriptions of *‘methylphenidate’* in US adolescents in recent years [30]. Choosing an appropriate time-period to study these kind of drugs is essential since the utilization of *‘neurological drugs’* and especially for the treatment of ADHD has changed tremendously since the start of VigiBase in 1968 [30, 32].

To our knowledge, our study is the first research focusing on long term use and on delayed adverse events in children. Describing of ICSRs reported after long-term drug use was a topic of special interest. Long-term drug use and long-term adverse events are of importance during childhood because of possible effects on growth and development. However, they are difficult to detect in clinical studies. These studies often lack sufficient time of follow-up and adverse events occurring long after initiating therapy are not easily recognised. Especially for drugs being used chronically or for adverse events that require a long duration of exposure (such as cancer, and certain types of infections), studies investigating long-term safety should be performed. The reported types of drugs before and after 6 months of use differed significantly. *‘Systemic hormonal preparations’*, *‘alimentary drugs’* and *‘antineoplastic/immunomodulating agents’* were prominently reported after long-term treatment (>6 months), while *‘anti-infective drugs’*, *‘musculoskeletal system drugs’* and *‘sensory organ drugs’* were reported mostly with short term use. These findings are in line with drugs known to be used short-term or are known to be used for long periods of time [29]. New onset neoplasms are an important concern and were more often reported after long-term drug use. It is not possible to infer any causal association based on spontaneous reporting data. However, the distribution of the drug classes and events reported after long-term drug use are in line with what is expected and therefore a complete dataset of pediatric ICSRs might be a suitable additional source to generate signals on delayed events and new onset chronic events.

The GRiP network aims to create an infrastructure promoting global drug safety surveillance in children. Signal detection analysis within spontaneous reporting databases is an important tool for pharmacovigilance, which may be followed by signal prioritization and evaluation analysis [24]. Information about the availability and the characteristics of data collected in systems as AERS is a key step in the development of pediatric specific methodology for post-marketing drug safety studies. Indeed, signal detection is influenced by the type of ICSRs, as previously demonstrated [33], as well as the distribution of other factors including the type of reporter and year of reporting [25]. For this reason, understanding the differences in the distribution of reported drugs and events within database and between different databases, like FAERS and VigiBase, gives insight on which factors might be of impact on the results but also helps choosing the right database for a specific research hypothesis.

The use of spontaneous reporting data has many well-known limitations [34]. Since the publically available datasets often do not include all variables there are analytic limitations and since case-narratives are lacking it is difficult to draw inferences on causality. For example, the non-availability of case-narratives implies a loss of potentially important information not otherwise coded in the ICSR. Another well-known, limitation is the volume of duplicates [35]. Duplicate reports are present in all spontaneous reporting databases [36]. The identification and elimination of duplicates from analyses is advantageous for using the data and is important for a correct interpretation of the data, however, so far, few easy to use duplicate-detection methods are currently available and enhanced methods of duplicate detection are being developed [35]. For this study, we dealt with the issue of duplicate reports by only including unique ICSRs. However it is inevitable that duplicate reports are still present within the used database.

## Conclusions

Knowledge on the distribution of the drug classes and events within FAERS is a key first step in developing pediatric specific methods for drug safety surveillance. Because of several differences in terms of drugs and events among age-categories, drug safety signal detection analysis in children need to be stratified by age groups.
